# Optimism and self-efficacy mediate the association between shyness and subjective well-being among Chinese working adults

**DOI:** 10.1371/journal.pone.0194559

**Published:** 2018-04-18

**Authors:** Conghui Liu, Ying Cheng, Anna S. C. Hsu, Chuansheng Chen, Jie Liu, Guoliang Yu

**Affiliations:** 1 Department of Psychology, Renmin University of China, Beijing, China; 2 Department of Psychology and Social Behavior, University of California Irvine, Irvine, California, United States of America; 3 The Students’ Psychological Development Service Center, Dalian Maritime University, Liaoning, Dalian, China; 4 School of Education, Renmin University of China, Beijing, China; Universita degli Studi di Firenze, ITALY

## Abstract

The aim of the current study was to investigate whether optimism and self-efficacy mediated the association between shyness and subjective well-being in a sample of Chinese working adults. Two hundred and eight participants completed the Revised Cheek and Buss Shyness Scale, Life Orientation Rest-Revised, Satisfaction with Life Scale, and Positive and Negative Affect Scale. Structural equation modeling results showed that optimism mediated the relationship between shyness and measures of subjective well-being (life satisfaction, positive and negative affect). Self-efficacy mediated the association between shyness and positive subjective well-being (life satisfaction and positive affect). These results suggest that optimism and self-efficacy play unique mediating roles in the relationship between shyness and subjective well-being. They also have important implications for the development of intervention programs aimed at promoting subjective well-being of Chinese working adults through enhancing self-efficacy and optimism.

## Introduction

Subjective well-being (SWB) is a predominant variable in the emerging field of positive psychology [1–3]. SWB includes two components: life satisfaction (i.e., cognitive evaluations) and negative or positive affect (affective evaluations) [2, 4–5]. Many studies have examined the association between shyness and the cognitive and affective components of SWB [6–11]. Results from these studies showed that shy individuals usually report more negative affect, less positive affect, and lower life satisfaction than individuals who are less shy.

Although shyness has been linked to SWB, few studies have assessed empirically the mediating mechanisms involved. Recently, several studies [11–13] found that the association between shyness and SWB was mediated by general self-efficacy (GSE), emotional intelligence (EI), and social support. Shy individuals were shown to have lower self-evaluations of their own abilities, lower emotional intelligence, and less perceived social support, which in turn, led to lower SWB.

Another likely mediator of the association between shyness and SWB is optimism or positive expectation about future events [14–15]. Individuals who are high in optimism have been shown to experience higher SWB than individuals who are low in optimism [16–19]. In addition, optimism has also been related to shyness [20]. Specifically, shy individuals are less optimistic, as reflected in negative tendencies in their attribution processes [21–23], and they perceive a lower level of controllability for both the self and the environment. More importantly, some studies showed that optimism partially mediated the relationship between meaning in life and SWB as well as the relationship between social support and SWB [24–25]. Thus, these results support the idea that optimism may mediate the relationship between shyness and SWB.

Although GSE and optimism may act similarly as mediators of the association between shyness and SWB, there are two important differences between GSE and optimism. One difference concerns the role of personal agency [15]. Self-efficacy is only one source of favorable expectancies for successful goal attainment, whereas optimism focuses on many situational factors (de-emphasizing the role of personal efficacy) including being in a positive environment, the availability of help from others, and a belief in the effectiveness of an action. The other difference concerns the scope of expectancy such that self-efficacy focuses on specific expectancy, whereas optimism focuses on generalized expectancy. Indeed, previous studies found that both specific and generalized expectancies made independent contributions to the prediction of individuals’ behaviors [19, 26].

The aim of the current study was to examine the mediating roles of optimism and general self-efficacy in the association between shyness and SWB and to see whether these two mediators made independent contributions. In contrast to the previous relevant studies that focused on college students in China [11, 13, 27–30], this study examined the mediation effects in a sample of Chinese working adults. It is crucial to examine these mediation effects with working adults because factors that affect the wellbeing of college students are different from those affecting adult employees and employees’ well-being is an important factor in their job performance [31]. By understanding of the mediation effects specific to adult employees, researchers and employers can develop more tailored intervention and training programs that aim to improve employees’ subjective well-being. Based on findings from previous studies, we proposed two hypotheses: (1) Shyness would be negatively associated with SWB and (2) Dispositional optimism and GSE would mediate the association between shyness and SWB.

## Method

### Participants

This study was approved by the Ethics Committee for Scientific Research of the Department of Psychology at Renmin University of China (April 15^th^, 2015). The participants were recruited through advertisements in WeChat Moments (a phone app) in early October, 2015. Two hundred and eight individuals (59 males and 149 females) who were at least 18 years old and working in various industries and types of companies (e.g., governmental agencies and institutions, nationalized business, foreign companies, and private companies) in Shanghai participated in our study. Information about participants is presented in Table 1. All participants signed the consent form for study participation before they took the survey.

**Table 1:** Participant information: Gender, age, education level, occupation, position and relationship status.

Category	Subcategory	Frequency	Percentage
Gender	Male	59	28.4
	Female	149	71.6
Age	18–25 years	29	13.9
	26–30 years	52	25.0
	31–40 years	84	40.4
	41–50 years	40	19.2
	> 50 years	3	1.4
Education	Finished high school	22	10.6
	College/University degree	141	67.8
	Graduate degree	45	21.6
Occupation (types of companies)	Governmental agencies and institutions	27	13.0
	Nationalized businesses	33	15.9
	Foreign companies	62	29.8
	Private companies	45	21.6
	Other	41	19.7
Occupation (types of positions)	Executives	17	8.2
	Mid-level managers	72	34.6
	Workers	87	41.8
	Other	32	15.4
Relationship Status	Single	70	33.7
	Married	126	60.6
	Divorced	10	4.8
	Remarried	2	1.0

### Measures

#### Revised Cheek and Buss Shyness Scale (RCBS)

The Revised Cheek and Buss Shyness Scale [32] has 13 items such as “I feel tense when I’m with people I don’t know” and “I am socially somewhat awkward.” Each item is rated on a 5-point Likert scale (1 = very uncharacteristic or untrue, 2 = uncharacteristic, 3 = neutral, 4 = characteristic, 5 = very characteristic or true). The RCBS has satisfactory reliability and validity. For example, the RCBS has been found to have high internal consistency (α = .90) and high 45-day, test-retest reliability (*r* = .88) [33]. In the current study, the internal consistency was .91.

#### General Self-Efficacy Scale (GSES)

The General Self-Efficacy Scale [34] has 10 items rated on a 4-point scale (1 = Not at all true, 2 = Hardly true, 3 = Moderately true, 4 = Exactly true). It assesses optimistic self-beliefs used to cope with various difficult demands in life. A sample item of this scale is “I can usually handle whatever comes my way”. The GSES has satisfactory reliability and validity [35], with high internal consistency (α = .87) and excellent 10-day, test-retest reliability (r = .83). The Cronbach alpha coefficient for the GSES in the current study was .91.

#### Life Orientation Test-Revised (LOT-R)

The LOT-R was designed by Scheier, Carver and Bridges [36] to assess dispositional optimism. The scale includes six statements (three positive items and three negative items) and four filler statements. A five-point scale (1 = strongly disagree, 2 = disagree a little, 3 = neither agree nor disagree, 4 = agree a lot, 5 = strongly agree) was used to rate items such as “I’m always optimistic about my future.” In the original study, the Cronbach’s alpha coefficient for the LOT-R was.74 [35]. In the current study, the Cronbach’s alpha coefficient was .65 for the 6-item LOT-R.

#### Satisfaction with Life Scale (SWLS)

The Satisfaction with Life Scale (SWLS), developed by Diener, Emmons, Larsen, and Griffin [37], consists of 5 brief statements. Respondents rated each of the five items on a 7-point scale (1 = strongly disagree, 2 = disagree, 3 = slightly disagree, 4 = neither agree nor disagree, 5 = slightly agree, 6 = agree, 7 = strongly agree). A sample item is ‘‘I am satisfied with my life”. Diener et al. [37] reported a Cronbach’s alpha of .87 for the scale and a 2-month test-retest stability coefficient of .82. In this study, the Cronbach’s alpha coefficient for the SWLS was .83.

#### Positive and Negative Affect Scale (PANAS)

The Positive and Negative Affect Scale developed by Watson, Clark, and Tellegen [38] consists of ten positive words describing positive affective (PA) state and ten negative words describing negative affective (NA) state. Participants are asked to indicate their general affect using a five-point scale (1 = very slightly or not at all, 2 = a little, 3 = moderately, 4 = quite a bit, 5 = extremely). The alpha reliabilities ranged from .86 to .90 for PA and from .84 to .87 for NA, and the test-retest stability coefficient was .68 for PA and .71 for PN on average [38]. In this study, the Cronbach’s alpha coefficient for the positive affect subscale was .88 and that for the negative affect subscale was .87.

### Procedure

Two hundred and eight working adults from various types of industries and companies in Shanghai voluntarily participated in the study. All participants were recruited via the WeChat Moments app on the internet. Each participant was asked to respond to measures of shyness, self-efficacy, optimism, and SWB (including positive affect, negative affect, and life satisfaction). Participants took an average of 20 minutes to complete all instruments included in the study. A step-by-step systematic review protocol was deposited in protocols.io(dx.doi.org/10.17504/protocols.io.mu5c6y6) [PROTOCOL DOI].

### Analytical strategy

We used a two-step procedure [39] to examine whether the pathways from shyness to life satisfaction, positive affect, and negative affect were mediated by optimism and self-efficacy. First, we tested the measurement model to evaluate the extent to which each of the latent variables was represented by its observed variables. We constructed parcels by randomly assigning items to parcels [40–41] to control for inflated measurement errors due to having multiple items for each latent variable [11, 29–30]. Three or four items were included in each parcel for the constructs of shyness, self-efficacy, positive affect, and negative affect. One or two items were included in each parcel for the construct of life satisfaction. Due to the unequal numbers of items in each parcel, we used the average scores of the items.

Once we obtained a satisfactory measurement model, we then examined the structural model using the maximum likelihood estimation in AMOS 17.0. As suggested by Schermelleh-Engel, Moosbrugger and Müller [42], we examined the chi-square statistic divided by the degrees of freedom to determine model fit. If the ratio is below 5, the model is considered to fit satisfactorily to the data [43]. We also used several other fit indices including the non-normed fit index (NNFI), comparative fit index (CFI), root mean square error of approximation (RMSEA), standardized root-mean-square residual (SRMR), Akaike’s Information Criterion (AIC), and expected cross-validation index (ECVI). Values greater than or equal to .90 for NNFI and CFI, indicate a good fit to the data [42, 44]. RMSEA and SRMR values that are less than or equal to .08 [44–45] also indicate an acceptable fit. For AIC and ECVI, a smaller value means a better fit [44, 46].

To assess the mediation effects, the Bootstrap estimation procedure in AMOS 17.0 (a bootstrap sample of 2000) was used. The bootstrap test is significant if both confidence limits have the same sign (e.g., both positive or both negative). This indicates that zero is not a likely value, and therefore, the null hypothesis of the indirect effect being zero should be rejected. As MacKinnon et al. [47] demonstrated, the bias-corrected bootstrap is the preferred method to obtain the most accurate confidence intervals for indirect effects. The main problem with traditional methods is that their estimates of indirect effects were based on the product of two effects, but the product of two normally distributed variables does not have a normal distribution [47].

## Results

### Measurement model

We evaluated the measurement model involving six latent variables (shyness, self-efficacy, optimism, positive affect, negative affect, and life satisfaction) and 18 observed variables. As shown in Table 2, all the latent variables were significantly correlated in conceptually expected ways (*p* < .001). An initial test of the measurement model generated a good fit to the data: *χ*^*2*^ (120, *N* = 208) = 238.30, *p* < .001; RMSEA = .07; SRMR = .06; NNFI = .93; CFI = .94. All the factor loadings for the indicators on the latent variables were significant (i.e., > .50, *p* < .001), which indicated that all the latent variables were well-represented by their observed variables.

**Table 2:** Descriptive statistics and zero-order correlations for measures.

	***Mean(SD)***	**1**	**2**	**3**	**4**	**5**	**6**
1. RCBS	2.48(0.74)	1					
2. GSES	3.03(0.52)	-.44***	1				
3. LOT-R	3.52(0.64)	-.63***	.61***	1			
4. SWLS	4.32(1.12)	-.31***	.38***	.54***	1		
5. PANAS-PA	3.41(0.65)	-.47***	.50***	.62***	.37***	1	
6. PANAS-NA	2.28(0.71)	.53***	-.31***	-.52***	-.36***	-.38***	1

RCBS = Revised Cheek and Buss Shyness Scale, GSES = General Self-Efficacy Scale, LOT-R = Life Orientation Test-Revised, PANAS-PA = Positive and Negative Affect Scale-Positive Affect, PANAS-NA = Positive and Negative Affect Scale-Negative Affect, SWLS = Satisfaction with Life Scale.

***p < .001.

### Structural model

Without the mediators in the model, the standardized coefficients’ paths from shyness to life satisfaction, positive affect, and negative affect were significant (*β* = -.35, *p* < .001; *β* = -.49, *p* < .001; *β* = .55, *p* < .001). The mediation model (S1 Fig) with self-efficacy and optimism as mediators showed a good fit to the data: ***χ***^***2***^ (124, *N* = 208) = 266.08, *p* < .001; RMSEA = .07; SRMR = .08; NNFI = .91; CFI = .93; AIC = 369.08; and ECVI = 1.91.

The paths from shyness to life satisfaction (*β* = .09, *p =* .51) and positive affect (*β* = -.08, *p =* .60) and that from self-efficacy to negative affect (*β* = .02, *p* = .85) to life satisfaction (*β* = 17; *p* = .08) were not significant in this mediation model.

The Bootstrap estimation procedure in AMOS 17.0 (a bootstrap sample of 2000) was used to assess the mediating effects of optimism and self-efficacy. The indirect effects and their 95% confidence intervals are shown in Table 3. Shyness had significant indirect effects on positive affect, negative affect, and life satisfaction via optimism; and significant indirect effects on positive affect and life satisfaction via self-efficacy. In other words, self-efficacy did not significantly mediate the associations between shyness and negative affect.

**Table 3:** Indirect effects and 95% confidence intervals.

Model pathways	Estimated	95% IC	
		Lower	Upper
Shyness→LOT-R→LS	^a^-.34	-.23	-.39
Shyness→LOT-R→PA	^a^-.29	-.19	-.36
Shyness→LOT-R→NA	^a^.22	.45	.07
Shyness→GSE→LS	^a^ -.08	-.01	-.12
Shyness→GSE→PA	^a^-.12	-.04	-.15
Shyness→GSE→NA	-.01	.08	-.06

GSE = general self-efficacy, LOT-R = life orientation test-revised, PA = positive affect, NA = negative affect, LS = life satisfaction

^a^ Empirical 95% confidence interval does not overlap with zero

## Discussion

The current study was conducted to investigate the mediating effects of self-efficacy and optimism on the relationship between shyness and SWB. The results showed significant relationships among shyness, self-efficacy, optimism, and SWB (life satisfaction, positive affect, and negative affect). These findings are consistent with earlier studies that reported significant relationships between shyness and self-efficacy [48], optimism [23], and SWB [6–11]. Likewise, previous researchers found significant relationships between SWB and self-efficacy [49–53], and between SWB and optimism [16–17, 19].

Also consistent with our hypothesis, results showed that the association between shyness and SWB was mediated by optimism and self-efficacy. First, optimism was a significant mediator between shyness and all three measures of SWB (life satisfaction, positive affect, and negative affect). Previous research [16] indicated that as a general positive evaluation of the self, environment, and self-environment interaction, optimism is a common predictor of both positive and negative components of SWB. It seems likely that shy individuals would hide from others or isolate themselves, which would lead them to feel more pessimistic, disappointed, and querulous about their life, which consequently reflect lower SWB. In contrast, people who are not shy take pleasure in communicating with other people, adopt a more positive outlook on life, and like to take on new challenges. Consequently, they have higher levels of SWB [21–25].

In contrast to optimism’s mediating role in the association between shyness and all three measures of SWB, self-efficacy was a significant mediator only between shyness and two measures of positive well-being (positive affect and life satisfaction). Previous research has shown that as a personal sense of competence, self-efficacy is a specific predictor of positive well-being [16, 54–56]. It seems that, because shy individuals usually have lower self-confidence (i.e. not believing in themselves) and have doubts about their ability to influence the future outcomes in important domains of life [11, 16], they are likely to have fewer achievement opportunities (a major source of positive well-being). In contrast, we found that self-efficacy was not a mediator between shyness and negative affect. This result is consistent with Zhang’s [57] recent finding that self-efficacy was not significantly correlated with negative affect. One explanation of this result is that self-efficacy motivates individuals to construct a positive perspective of life, independent of their levels of optimism, but self-efficacy’s contribution to negative affect (i.e., their significant bivariate association) was shared with that of optimism. In other words, self-efficacy did not serve as a significant and unique mediator between shyness and negative affect after optimism was entered into model 1.

The current study has several limitations. First, our study relied on correlations, so we could not draw a causal relationship among the variables. Second, the data in this study were only collected via participants’ self-reports, which may be affected by social desirability. In addition, some associations may have resulted from common source variance. Other-reports (e.g., parent and peer report) or implicit measures should be used in future research. Third, the sample was drawn from various occupations in China. It is unknown whether the same results can be replicated with participants from other cultural backgrounds (especially Western participants). As a result, the generalizability of our findings (particularly to other cultural groups) may be limited.

Despite these limitations, the current study provided meaningful evidence for the mediating mechanisms between shyness and SWB. The results from a sample of Chinese working adults suggest that people who are shy usually are less optimistic and have lower levels of self-confidence, which in turn would contribute to lower SWB. The findings in the current study provide valuable information and guidance for successful implementations of psychological interventions or educational programs aimed at increasing the well-being of shy individuals. According to our findings, it is likely that using specific strategies and techniques for enhancing optimism (which is associated with both positive and negative aspects of SWB) and improving self-efficacy (which is related to positive aspects of SWB) may lead to more effective psychological interventions and programs aimed at increasing SWB.

## Supporting information

S1 Fig Model 1 (N = 208). Factor loadings are standardized. Shyness_1-Shyness_3 = three parcels of shyness; GSE_1-GSE_3 = three parcels of general self-efficacy; LOT_R_1-LOT_R_3 = three parcels of optimism; PA_1-PA_3 = three parcels of positive affect; NA_1-NA_3 = three parcels of negative affect; LS_1-LS_3 = three parcels of life satisfaction. ***p < .001, **p < .01, p*< .05, *p*^†^< .10.
(TIF)

S1 Table Participant-level data. All reverse variables have been reverse coded.
(XLSX)

S1 Protocol Institutional review board notice of approval. (IRB Protocol Number:15–102).
(PDF)
