# Supplementary figures and images for: Optimism and self-efficacy mediate the association between shyness and subjective well-being among Chinese working adults
Source: PLoS One. 2018 Apr 18;13(4):e0194559. doi: 10.1371/journal.pone.0194559 (PMC5905885; doi:10.1371/journal.pone.0194559)

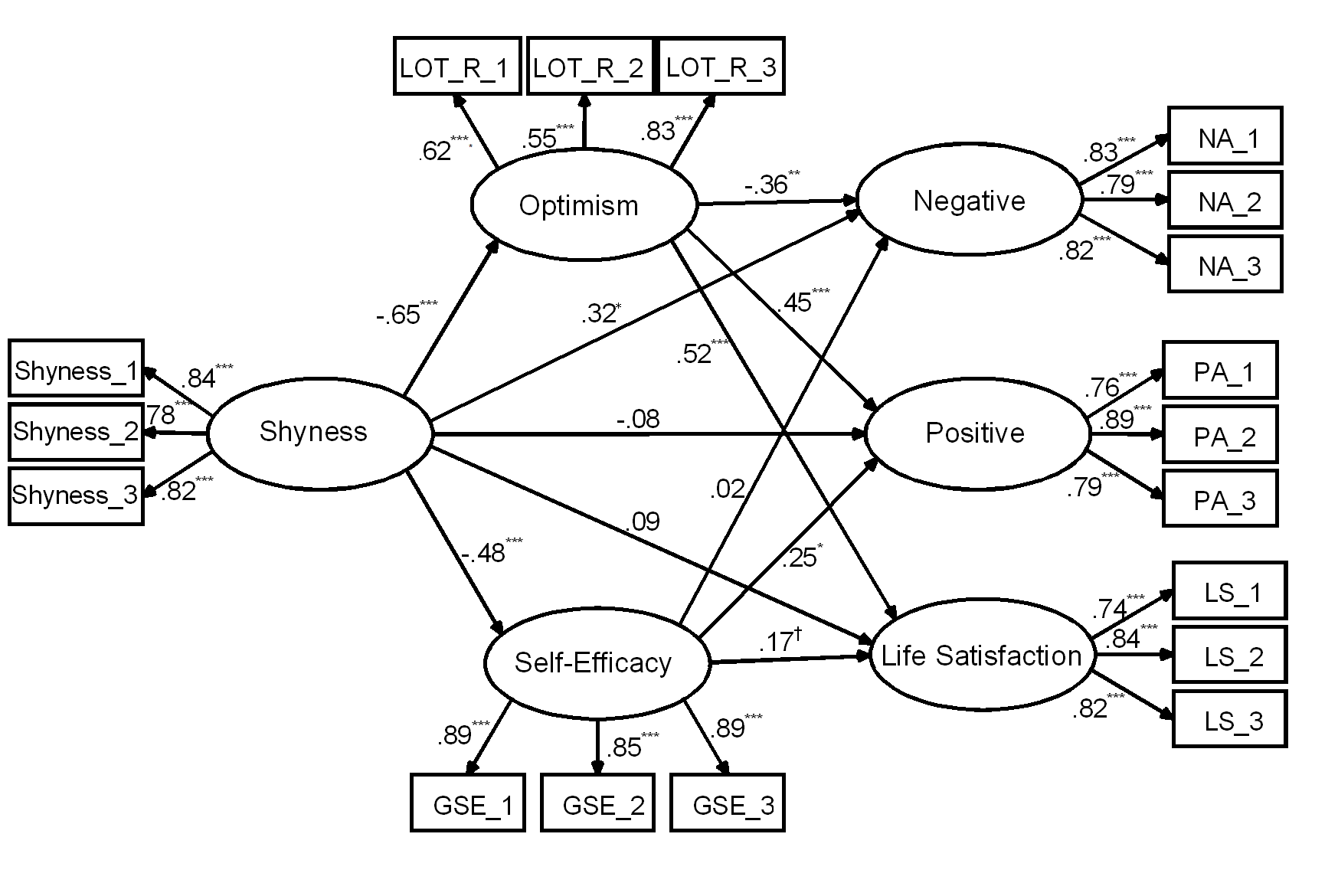

Supplement: S1 Fig — Factor loadings are standardized. Shyness_1-Shyness_3 = three parcels of shyness; GSE_1-GSE_3 = three parcels of general self-efficacy; LOT_R_1-LOT_R_3 = three parcels of optimism; PA_1-PA_3 = three parcels of positive affect; NA_1-NA_3 = three parcels of negative affect; LS_1-LS_3 = three parcels of life satisfaction. ***p < .001, **p < .01, p*< .05, p†< .10. (TIF) [file pone.0194559.s001.tif]
