# Supplementary material for: Optimism and self-efficacy mediate the association between shyness and subjective well-being among Chinese working adults
Source: PLoS One. 2018 Apr 18;13(4):e0194559. doi: 10.1371/journal.pone.0194559 (PMC5905885; doi:10.1371/journal.pone.0194559)
Supplement: S1 Protocol — (IRB Protocol Number:15–102). (PDF) [file pone.0194559.s003.pdf]

Office of regulatory research compliance

Renmin University of China

Institution Review Board

Notice of Approval

IRB Protocol Number: 15-102

Date:April 7,2015

Principal investigator: **Conghui Liu**

Title: **Studies of well-being**

Review Type:☐Full Committee☒Expedited   Expedited Review Category#7

Approval Type:☒Yearly Approval Continuation   Continuation#1

**Approval Date:**	**April15,2015**	**Expiration Date:**	**April15,2016**	**Review Cycle:**	**1 Year**

1. **Provisions of Approval:n/a**
2. **Consent Forms:**All participants must receive a copy of the consent form as approved with the RenminUniversity of China Institution Review Board stamp.Copies of the signed consent form must be kept on file.
3. **Research Records:**Accurate and detailed research records must be maintained.All research records(including all IRB correspondence)must be kept for a minimum of 3 year after the completion of the research.
4. **Changes:**No changes in the above referenced study may be initiated without prior IRB review and approval.Changes include(but are not limited to)study personnel,consent forms,protocol,procedures,addition of funding source.
5. **Lapse of Approval:**If approval for this project lapses, all research must stop **IMMEDIATELY** until continuation approval is granted.If approval lapses for longer than 30 days,your project must be resubmitted as a new protocol.
6. **Yearly IRB Approval Continuation:**Approval is valid until the expiration date above.You are required to obtain annual IRB approval continuations prior to your expiration date for as long as the study is active.An annual continuation reminder will be sent to you,but it is your responsibility to ensure that you submit and receive the yearly approval in a timely manner.Up to 4 yearly continuations will be granted after which a new protocol must be submitted for review.
7. **Funded Research:** If your research is funded,you must also submit sponsor information and two copies of the grant/funding application for IRB review with the human subjects section(s) highlighted. This is true whether the source of funding is internal or external.
8. **University Permissions:** A)Institutional Research,Planning and Effectiveness(IRPE)permission may be required if your research participants are recruited fromRenmin University of China.It is the responsibility of the investigator to contact IRPE for a determination.B)All permissions(e.g.classroom,team or organization permissions)must be kept on file with your research records.
9. **Posters or Flyers:** If posters of flyers are to be posted on the campus ofRenmin University of China,they must be registered with the Office of Student Involvement and Leadership prior to posting on the academic Podium.
10. **External Permissions:**All external permissions (e.g.schools,businesses,organizations,etc.)must be kept on file with your search records.

The IRB wishes your success with your research.

Approved by IRB
Valid Thru:
15 APR 2016
The Department of Psychology RUC

Yongna Li

Assistant Vice President for Research

Director/Research Compliance Officer

On behalf of the institutional Review Board

Cc: Conghui Liu

Department of Psychology, Renmin University of China

No.59, Zhongguancun Street, Haidian District, Beijing, China

Postal code: 100872Tel: 86-10-82509716

Fax: 86-10-82509716E-mail: liuconghui2001@ruc.edu.cn
